# As(V) Sorption/Desorption on Different Waste Materials and Soil Samples

**DOI:** 10.3390/ijerph14070803

**Published:** 2017-07-19

**Authors:** Ana Quintáns-Fondo, David Fernández-Calviño, Juan Carlos Nóvoa-Muñoz, Manuel Arias-Estévez, María J. Fernández-Sanjurjo, Esperanza Álvarez-Rodríguez, Avelino Núñez-Delgado

**Affiliations:** 1 Department of Soil Science and Agricultural Chemistry, Engineering Polytechnic School, University of Santiago de Compostela, 27002 Lugo, Spain; anaquintansfondo@hotmail.com (A.Q.-F.); mf.sanjurjo@usc.es (M.J.F.-S.); esperanza.alvarez@usc.es (E.Á.-R.); 2 Department of Plant Biology and Soil Science, Faculty of Sciences, Campus Ourense, University of Vigo, 32004 Ourense, Spain; davidfc@uvigo.es (D.F.-C.); edjuanca@uvigo.es (J.C.N.-M.); mastevez@uvigo.es (M.A.-E.)

**Keywords:** arsenic retention/release, hemp waste, mussel shell, oak ash, pine bark

## Abstract

Aiming to investigate the efficacy of different materials as bio-sorbents for the purification of As-polluted waters, batch-type experiments were employed to study As(V) sorption and desorption on oak ash, pine bark, hemp waste, mussel shell, pyritic material, and soil samples, as a function of the As(V) concentration added. Pyritic material and oak ash showed high sorption (90% and >87%) and low desorption (<2% and <7%). Alternatively, hemp waste showed low retention (16% sorption and 100% desorption of the amount previously sorbed), fine shell and pine bark sorbed <3% and desorbed 100%, the vineyard soil sample sorbed 8% and released 85%, and the forest soil sample sorbed 32% and desorbed 38%. Sorption data fitted well to the Langmuir and Freundlich models in the case of both soil samples and the pyritic material, but only to the Freundlich equation in the case of the various by-products. These results indicate that the pyritic material and oak ash can be considered efficient As(V) sorbents (thus, useful in remediation of contaminated sites and removal of that pollutant), even when As(V) concentrations up to 6 mmol L^−1^ are added, while the other materials that were tested cannot retain or remove As(V) from polluted media.

## 1. Introduction

Arsenic pollution is a public hazard, mainly in relation to drinking water. As(V) species are predominant in aerated surface waters, whereas the presence of As(III) is more prevalent in groundwater. Forest areas can suffer arsenic pollution due to wood preservative compounds that include As [1], and vineyard soils can be affected by As-based herbicides [2], resulting in increased risks of pollution [3].

Although different methods can be used to remove As from liquid media, adsorption has been considered as a relatively simplistic and safer alternative [4]. Previous studies have focused on As retention and release on soils and different sorbent materials [5,6,7], and the use of bio-sorbents has been considered as a low-cost alternative to remove As from polluted media, similar to that showed for other pollutants [8,9]. In fact, many low-cost sorbents have been previously investigated regarding their As removal potential, some of them showing encouraging results [10]. We have previously studied As(V) retention on soils and wastes [11,12,13,14,15,16,17], using low As(V) concentrations (<1.5 mmol L^−1^). In recent works, we used different sorbent materials to study As(V) and Cr(VI) competitive sorption [18,19], but non-competitive As(V) sorption/release was not investigated.

As indicated by Núñez-Delgado et al. [20], for As(V)-polluted media, it would be interesting to determine the retention potential of sorbent materials candidate to remove As(V), covering a wide As(V)-concentrations interval.

In view of that, the objective of the present work is to investigate non-competitive As(V) sorption/desorption on oak ash, hemp waste, pine bark, fine mussel shell, pyritic material, and on forest and vineyard soil samples, for different As(V) concentrations added (up to 6 mmol L^−1^). The results of this research could aid in programming the appropriate management of the studied soils and waste materials when focusing on As(V) retention and/or removal, thus aiding to fight As(V) water pollution.

## 2. Materials and Methods

### 2.1. Materials

We studied the following materials: oak ash, hemp waste, pine bark, finely ground mussel shell, pyritic material, forest and vineyard soil samples. The forest and vineyard soil, as well as the mussel shell samples were previously described [13], as the oak ash and the hemp waste [18], the pyritic material [21], and the pine bark [22]. Furthermore, Rivas-Pérez et al. [17] previously studied some of the materials here assayed.

Full details regarding analytical methods [23,24,25,26,27,28,29,30,31,32,33,34,35,36,37,38,39,40,41,42,43,44,45] and results corresponding to chemical characteristics of each material are included in the Supplementary Materials (see Table S1, as well as Figures S1–S7).

### 2.2. Methods

#### 2.2.1. Sorption/Desorption Experiments

As(V) sorption/desorption experiments were performed as per Arnesen and Krogstrad [46], working at 20 ± 1 °C.

To carry out the procedure, individual samples (3 g of each soil or waste material) were added with 30 mL of 0.01 M NaNO_3_ dissolutions containing 0, 0.5, 1.5, 3, and 6 mmol As(V) L^−1^, prepared from analytical grade Na_2_HAsO_4_ (Panreac, Barcelona, Spain). Each suspension was shaken for 24 h, centrifuged for 15 min (6167× *g*), and filtered through acid-washed paper (2.5 µm pore size). The following parameters were determined in the equilibrium dissolutions: pH (glass electrode, Crison, Barcelona, Spain), dissolved organic carbon (DOC) (UV-visible spectroscopy -UV-1201, Shimadzu, Kyoto, Japan), and As (ICP-mass -Varian 800-NS, Denver, CO, USA). Sorbed As(V) was estimated as the difference between the concentration of As(V) added and As remaining in the equilibrium solution.

After the end of the sorption trials, 30 mL of 0.01 M NaNO_3_ were added to each individual sample to desorb As(V). They were then shaken, centrifuged and filtered as above, and desorbed As, DOC and pH were quantified. The difference between the concentration retained in the sorption phase and that released in the desorption phase, was used to calculate desorbed As, which was expressed as a percentage of the concentration sorbed.

Triplicate trials were carried out for each of the sorption and desorption tests.

#### 2.2.2. Data Analyses

Statistical analyses were carried out by means of SPSS 21 (IBM, New York, NY, USA). Specifically, it was used for descriptive statistics, stepwise linear regression and correlation analyses, considering statistical significance for *p* < 0.05. It was also used to fit As sorption data to the Freundlich and Langmuir models.

The Freundlich equation is formulated as:

q_e_ = K_F_·C_e_^n^ (1)

with q_e_ being the As(V) sorbed per unit of mass of the sorbent, C_e_ the equilibrium concentration of the dissolved As, K_F_ a constant related to the sorption capacity, and n a constant related to the sorption intensity.

The Langmuir equation is formulated as:

q_e_ = X_m_·K_L_·C_e_/(1 + K_L_·C_e_) (2)

with X_m_ being the maximum sorption capacity and K_L_ a constant related to the sorption energy.

## 3. Results

### 3.1. As Sorption for Different As(V) Concentrations Added

**Figure 1:**
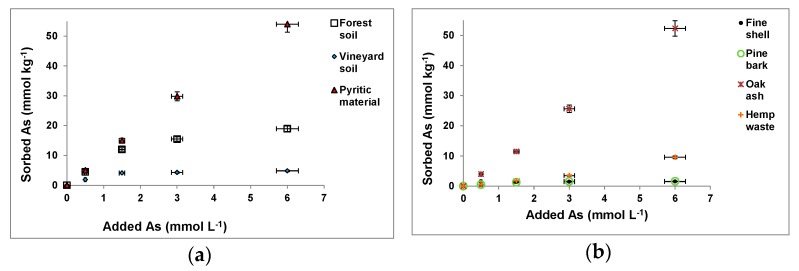
As(V) sorption for different As(V) concentrations added to the soil and pyritic samples (**a**) and to the waste materials (**b**) tested. Mean values for 3 replicates, with error bars (coefficients of variation always <5%).

As(V) sorption results corresponding to the forest and vineyard soil samples, pyritic material, and the other waste materials, for different As(V) concentrations added, are shown in Figure 1.

For the forest and vineyard soil samples, mussel shell and pine bark, As(V) sorption clearly increased, meanwhile, added As(V) was 1.5 mmol L^−1^ or lower, then showing a much lower sorption increase (Figure 1a,b). However, the pyritic material, oak ash, and hemp waste showed increased As(V) sorption for all As(V) concentrations added.

Osorio-López et al. [12], Seco-Reigosa et al. [16], and Rivas-Pérez et al. [17] also showed increased As(V) sorption as a function of the As(V) concentration added for most of the soil and waste samples here used, although clearly lower As(V) concentrations (<1.3 mmol L^−1^) were added in these previous studies.

Figure 1a,b show that maximum As(V) sorption when 6 mmol As(V) L^−1^ were added corresponded to the pyritic material (54 mmol kg^−1^, representing 90% of the As(V) added), followed by oak ash (52.29 mmol kg^−1^, 87% of added As(V)), forest soil sample (32% of added As(V)), hemp waste (16% of added As(V)), vineyard soil sample (8% of added As(V)), and pine bark and fine mussel shell (both 3% of the As(V) added).

Taking into account that pointed out by Liu and Zhang [47], the high As(V) sorption on the pyritic material may be due to the fact that at the acid pH prevailing the dominant As(V) species is H_2_AsO_4_^−^, which could sorb on positively charged Fe and Al sites on the pyritic material (Table S1, Supplementary Materials). Oak ash also showed high As(V) sorption, although having alkaline pH, situation where HAsO_4_^2−^ is dominant [47], and the variable charge compounds are not protonated. In fact, these ashes have notable concentrations of Fe_o_ and Al_o_ (Table S1, Supplementary Materials), which are negatively charged at alkaline pH, favoring sorption of anionic As(V) by means of cationic bridges. In the case of the forest soil sample, sorption was 90% when 0.5 mmol L^−1^ As(V) was added, decreasing to 80% when the added concentration was 1.5 mmol L^−1^, which was similar to that previously found by Osorio-López et al. [12]. However, in the present study, much higher As(V) concentrations were added, which caused clearly lower sorption results (33% when a 6 mmol As(V) L^−1^ concentration was added). The vineyard soil, fine shell, and pine bark samples also showed a progressive decrease in percentage sorption as As(V) concentration added increased, indicating progressive saturation of sorption sites when high As(V) concentrations are present. See below further comments regarding pH evolution in the equilibrium solution as a function of As(V) concentration added, which could affect sorption.

The pyritic material showed the highest As(V) sorption (always >90%), and also high in the case of the oak ash (always >76%, and >87% when 6 mmol As(V) L^−1^ was added). Taking into account that the pyritic material showed a pH of 2.97, and that it was 11.31 for oak ash, this fact was in line with the wide pH range (4–11) signaled by Stanic et al. [48] for As(V) sorption on zeolites. It was also coincident with findings by Williams [49], and Smedley and Kinniburgh [50], although differing in narrower ranges found for impregnated alumina (6–8) [51], or for impregnated chitosan (2–4) [52].

For the forest soil sample, sorption was relevant (>80%) only when added As(V) concentrations were low (1.5 mmol L^−1^ or lower), which can be related to the presence of organo-aluminum complexes.

As(V) sorption was low on hemp waste (<16%), pine bark (<9.9%), vineyard soil sample (<39%), and fine mussel shell (<31%). The latter has alkaline pH, with HAsO_4_^2−^ being dominant [47,53], which can bind to calcite (abundant in mussel shell) by inner sphere complex with octahedral Ca [54], although carbonates have much lower As(V) sorption capacity than Fe oxides [55].

Regarding pH evolution in the equilibrium solution, it increased as a function of increased As(V) sorption for forest soil sample (from 3.62 to 5.5), vineyard soil sample (from 3.84 to 6.02), pyritic material (from 3.07 to 3.61), and fine mussel shell (from 6.40 to 7.97), which can be related to anionic exchange of As(V) species and OH^−^ groups [46,56]. This effect could have influenced adsorption, especially for materials with low buffer abilities, such as the pyritic residue. For the pyritic material, a significant correlation was found between pH in the equilibrium and sorbed As(V) for each As(V) concentration added (r = 0.968, *p* < 0.01). However, pH remained almost unchanged in the case of pine bark (3.46–3.74), oak ash (13.11–13.13), and hemp waste (8.37–8.25), suggesting the absence of anionic exchange with OH^−^ in these materials.

However, other possible As(V) sorption mechanisms do not cause release of OH^−^ [57], and it is also possible that anionic exchange processes cause SO_4_^2−^, PO_4_^3−^, or organic anions release. In relation to the latter, DOC (mg L^−1^) increased in the equilibrium solution as a function of As(V) sorption for the forest soil sample (from 5.5 to 42.4), vineyard soil sample (6.9 to 32.4), pyritic material (0.7 to 2.0), fine shell (6.3 to 11.6), pine bark (15.9 to 23.7), oak ash (92.0 to 102.0), and hemp waste (from 383.4 to 491.7). Significant correlations were found between sorbed As(V) and DOC for forest soil sample (r = 0.908, *p* < 0.05), pyritic material (r = 0.934, *p* < 0.05), and hemp waste (r = 0.894 *p* < 0.05), suggesting a release of organic anions during As(V) sorption [58,59].

### 3.2. As Sorption Curves

**Figure 2:**
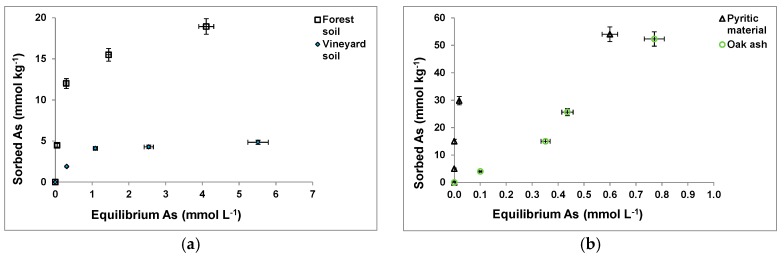

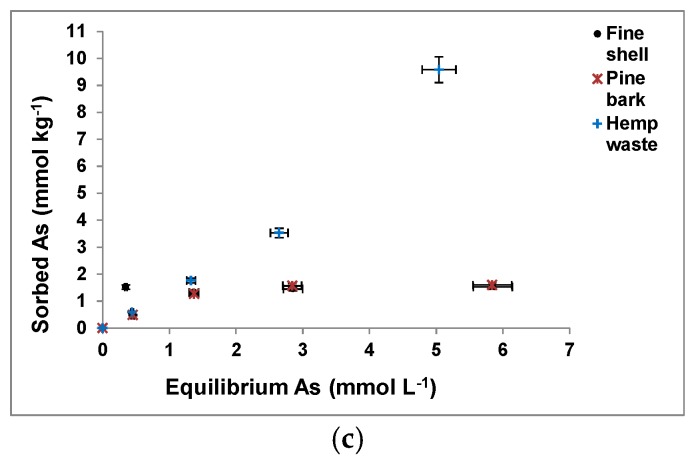
As(V) sorption curves for the soil samples and waste materials tested (**a**–**c**). Mean values for 3 replicates, with error bars (coefficients of variation always <5%).

Figure 2 shows As(V) sorption curves for the soil samples (Figure 2a) and waste materials assayed (Figure 2b,c), showing a clearly higher sorption on the pyritic material and the oak ash samples (Figure 2b), followed by the forest soil sample (Figure 2a) and the hemp waste (Figure 2c).

Table 1 shows that As(V) sorption presented good adjustment to the Langmuir and Freundlich models for both soil samples and the pyritic material, but fine shell, pine bark, oak ash, and hemp waste cannot be adjusted to the Langmuir model. Maji et al. [60] found good fitting to both Langmuir and Freundlich models when studying As(V) sorption on lateritic soils, whereas Yolcubal and Akyol [61] found better adjustment to the Freundlich equation for As sorption on carbonate-rich soils.

Although utilizable data regarding the Langmuir model was too scarce, it can be commented that a positive correlation was found between Langmuir’s K_L_ and Fe_T_ (r = 0.950, *p* < 0.01) and Fe_o_ (r = 0.991, *p* < 0.01), as well as between Langmuir’s X_m_ and Al_o_ (r = 0.858, *p* < 0.05). This can be due to the influence of amorphous Fe and Al compounds on As(V) sorption. In addition, positive correlations were also found between K_L_ and Al saturation (r = 0.847, *p* < 0.05), and between X_m_ and Mg_e_ (r = 0.923, *p* < 0.01), Na_e_ (r = 0.954, *p* < 0.01), K_e_ (r = 0.987, *p* < 0.01), eCEC (r = 0.970, *p* < 0.01), Mg_T_ (r = 0.960, *p* < 0.01), and K_T_ (r = 9.991, *p* < 0.01).

Regarding the Freundlich constants, K_F_ correlated with Mg_T_ (r = 0.851, *p* < 0.05) and Mn_T_ (r = 0.771, *p* < 0.05), and the n parameter correlated with Mg_e_ (r = 0.878, *p* < 0.01), K_e_ (r = 0.773, *p* < 0.05), eCEC (r = 0.827, *p* < 0.05) and K_T_ (r = 0.762, *p* < 0.05). These correlations can be in relation to As(V) binding through cationic bridges in some cases. Furthermore, some of these cations can be in the form of oxides, thus favoring surface bindings [62].

### 3.3. As(V) Desorption

**Figure 3:**
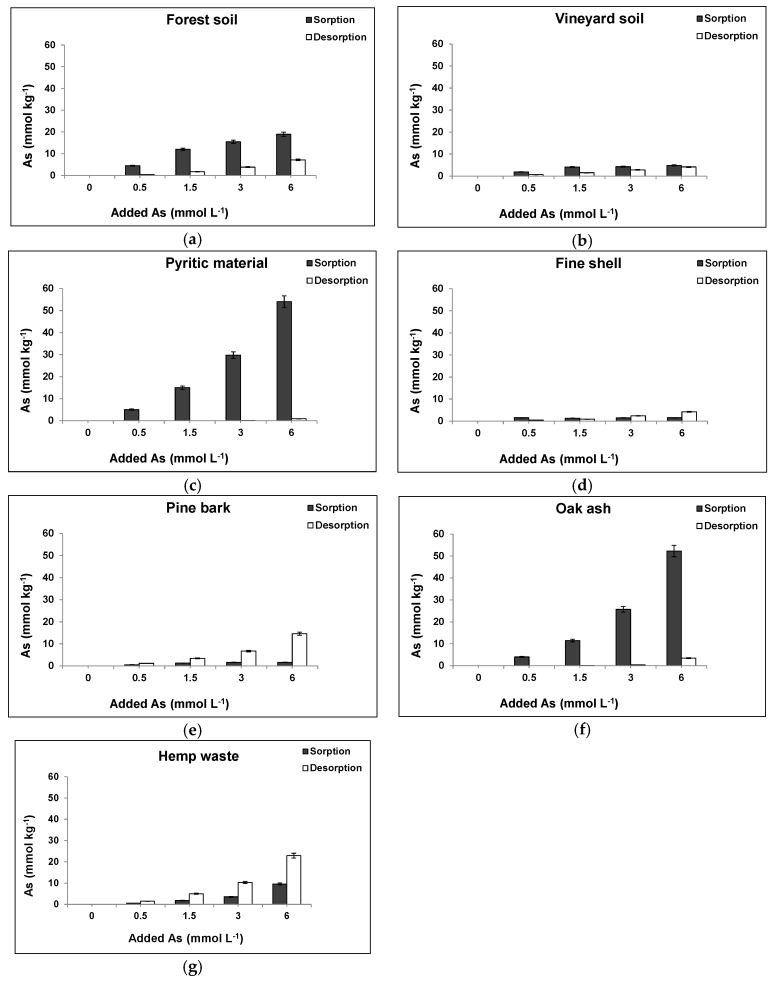
As(V) sorption and desorption for each soil sample and waste material and different As(V) concentrations added. Mean values for 3 replicates, with error bars (coefficients of variation always <5%). (**a**) Forest soil; (**b**) Vineyard soil; (**c**) Pyritic material; (**d**) Fine mussel shell; (**e**) Pine bark; (**f**) Oak ash; (**g**) Hemp waste.

Figure 3 shows As(V) desorption and sorption for each material and for any As(V) concentration added, showing that forest soil sample released 38% of the As(V) previously sorbed when 6 mmol As(V) L^−1^ was added (Figure 3a). Desorption was clearly higher from the vineyard soil sample (85%) (Figure 3b), and even higher from the fine shell, pine bark, and hemp waste (100%) (Figure 3d,e,g). However, the pyritic material desorbed just 1.6% (Figure 3c), and oak ash desorbed 6.5% (Figure 3f), indicating a notable degree of irreversibility in the sorption process taking place on both materials.

Strong As(V) bonds on Fe oxides at acid pH could explain its low release from the pyritic material, showing that pH reaches alkalinity to significantly increase desorption [55]. For oak ash, low As(V) release can be due to the high cation concentrations characterizing this material (Table S1, Supplementary Materials), including those in oxides which can retain As(V), as previously noted by Rahman et al. [62] studying wood ash.

## 4. Conclusions

The pyritic material and the oak ash here studied can be considered effective As(V) sorbents, in view of their high sorption and low desorption capabilities. However, the studied hemp waste, fine shell, pine bark, and the vineyard soil sample did not act as appropriate bio-sorbents for As(V) removal, whereas the forest soil sample showed slightly better results. Sorption data adjusted better to the Freunlich than to the Langmuir model, indicating a low probability of easy saturation of sorption sites. These results indicate that oak ash and the pyritic material are the best As(V) sorbents among those here studied, being useful to fight As(V) pollution, even against As(V) concentrations up to 6 mmol L^−1^.

## Figures and Tables

**Table 1:** Constants and R^2−^coefficients for fitting of As(V) sorption data to the Freundlich and Langmuir models in the soil samples and waste materials studied. Error values into brackets. -: too high error values avoid fitting.

Sorbent Material	Freundlich			Langmuir		
	K_F_ (L^n^ kg^−1^ mmol^(1−n)^)	n	R^2^	K_L_ (L mmol^−1^)	X_m_ (mmol kg^−1^)	R^2^
Forest soil	13.70 (±0.89)	0.25 (±0.05)	0.97	5.68 (±1.22)	18.74 (±0.85)	0.99
Vineyard soil	3.33 (±0.33)	0.25 (±0.08)	0.95	2.17 (±0.55)	5.28 (±0.31)	0.99
Pyritic material	58.01 (±3.60)	0.16 (±0.02)	0.99	62.96 (±49.84)	55.31 (±9.70)	0.87
Fine shell	1.46 (±0.05)	0.020 (±0.00)	0.99	-	-	-
Pine bark	0.97 (±0.15)	0.34 (±0.12)	0.92	-	-	-
Oak ash	78.32 (±7.93)	1.53 (±0.21)	0.98	-	-	-
Hemp waste	0.98 (±0.15)	1.40 (±0.10)	1.00	-	-	-

K_F_: Sorption capacity constant; n: Sorption intensity constant; K_L_: Sorption energy constant; X_m_: Maximum sorption capacity; R^2^: Coefficient of determination.

## Supplementary Materials

The following are available online at www.mdpi.com/1660-4601/14/7/803/s1, Table S1: General characteristics of the sorbent materials (average values for 3 replicates, with coefficients of variation always <5%), Figure S1: Infrared spectrum of forest soil, Figure S2: Infrared spectrum of vineyard soil, Figure S3: Infrared spectrum of pyritic material, Figure S4: Infrared spectrum of fine mussel shell, Figure S5: Infrared spectrum of pine bark, Figure S6: Infrared spectrum of oak ash, Figure S7: Infrared spectrum of hemp waste.
