# Supplementary material for: As(V) Sorption/Desorption on Different Waste Materials and Soil Samples
Source: Int J Environ Res Public Health. 2017 Jul 19;14(7):803. doi: 10.3390/ijerph14070803 (PMC5551241; doi:10.3390/ijerph14070803)
Supplement: Supplementary file 1 [file ijerph-14-00803-s001.pdf]

Supplementary Materials: As(V) Sorption/Desorption on Different by Products and Soil Samples

Characterization of the Materials

The forest soil samples were from an A horizon in a soil developed over granitic rocks near the Alcoa aluminum factory (San Cibrao, Lugo Province, Spain). The vineyard soil was developed over schists, and it was sampled in Sober (Lugo Province, Spain). The pyritic material was from a copper mine tailing (Touro, A Coruña Province, Spain). The finely ground (<1 mm) mussel shells were from the Abonomar S.L. factory (A Illa de Arousa, Pontevedra Province, Spain). The pine bark was a commercial product from Geolia (Madrid, Spain), where the <0.63 mm particle size fraction was used after grounding and sieving. The oak ash was from a combustion boiler in Lugo (Spain). The hemp waste, with particle size between 0.63–5 mm, was from an enterprise working on hemp-derived products, situated in Guitiriz (Lugo Province, Spain).

The forest soil, the vineyard soil, and the pyritic material were sampled at 0–20 cm depth in a zigzag manner (10 subsamples taken to perform each final composite sample). All these samples were air dried and sieved through 2 mm in the laboratory, and chemical determinations were carried out on the <2 mm fraction, with all determinations being performed in triplicate.

C and N were determined on 5 g samples by means of an elemental Tru Spec CHNS auto-analyzer (LECO Corporation, St. Joseph, MI, USA) (Chatterjee et al., 2009). pH in water was measured on 10 g of solid sample (solid: liquid relation 1 : 2.5) using a pH-meter (model 2001, Crison, L'Hospitalet de Llobregat, Barcelona, Spain) (McLean, 1982), also used to measure the point of zero charge (pH_{pzc}) (Mimura et al., 2010). Exchangeable Ca, Mg, Al, Na and K were quantified by atomic absorption and emission spectroscopy (AAnalyst 200, Perkin Elmer, Boston, MA, USA) after extraction from 5 g samples with a 1 M NH_4Cl solution (Sumner and Miller, 1996); the effective cationic exchange capacity (eCEC) was calculated as the sum of these cations (Kamprath, 1970). Total P was quantified on 1 g samples using UV-visible spectroscopy (UV-1201, Shimadzu, Kioto, Japan) after nitric acid (65%) microwave assisted digestion (Tan, 1996). To determine total concentrations of Na, K, Ca, Mg, Al, Fe, Mn, As, Cd, Cr, Cu, Ni and Zn, a nitric acid (65%) microwave assisted digestion was carried out on 1 g samples, then quantifying by means of ICP-mass spectrometry (820-NS, Varian, Palo Alto, CA, USA) (Nóbrega et al., 2012). Total concentrations of non-crystalline Al and Fe (Al_o, Fe_o) were measured using ammonium oxalate solutions (acidified to pH 3 with oxalic acid) on 1 g samples (Álvarez et al., 2012). All trials were performed by triplicate. Table S1 shows the results corresponding to the chemical characterization of the materials assayed. In addition, the particle-size distribution of forest and vineyard soil samples was determined by using the Robinson pipette procedure, giving: forest soil 65% sand, 20% silt and 15% clay; vineyard soil 73% sand, 12% silt and 15% clay.

Table S1. General characteristics of the sorbent materials (average values for 3 replicates, with coefficients of variation always <5%).

	**Forest soil**	**Vineyard soil**	**Pyritic material**	**Fine shell**	**Pine bark**	**Oak ash**	**Hemp waste**
C (%)	4.22	2.94	0.26	11.43	46.95	11.65	36.53
N (%)	0.33	0.23	0.04	0.21	0.32	0.21	2.81
pH_{water}	5.65	4.48	2.97	9.39	3.99	11.31	8.70
pH_{pzc}	5.53	4.14	3.46	9.94	4.00	12.52	9.00
Ca_e ($cmol_{(+)}$ kg^{-1})	4.37	1.78	0.36	24.75	5.38	95.03	31.15
Mg_e ($cmol_{(+)}$ kg^{-1})	0.66	0.24	0.29	0.72	2.70	3.26	3.67
Na_e ($cmol_{(+)}$ kg^{-1})	0.33	0.14	0.14	4.37	0.46	12.17	4.19
K_e ($cmol_{(+)}$ kg^{-1})	0.60	0.83	0.24	0.38	4.60	250.7	21.82
Al_e ($cmol_{(+)}$ kg^{-1})	1.92	2.28	2.86	0.03	1.78	0.07	<0.001
eCEC ($cmol_{(+)}$ kg^{-1})	7.88	5.27	3.89	30.25	14.92	361.2	60.83
P_T (mg kg^{-1})	423.9	679.3	606.3	101.5	<0.01	663.7	1935
Ca_T (mg kg^{-1})	708.5	607.1	603.0	280168	2319	136044	13258
Mg_T (mg kg^{-1})	830.5	5003	8384	980.6	473.6	26171	6987
Na_T (mg kg^{-1})	515.1	297.6	412.0	5173	68.92	2950	663.0
K_T (mg kg^{-1})	1544	5441	3186	202.1	737.8	99515	10438
As_T (mg kg^{-1})	4.18	3.41	7.0	1.12	<0.001	8.36	0.76
Cd_T (mg kg^{-1})	0.43	0.14	0.08	0.07	0.13	19.93	0.08
Cr_T (mg kg^{-1})	18.35	41.44	99.0	4.51	1.88	36.28	8.66
Cu_T (mg kg^{-1})	15.72	521.1	773.0	6.72	<0.001	146.33	18.06
Ni_T (mg kg^{-1})	10.69	21.73	5.0	8.16	1.86	69.25	8.03
Zn_T (mg kg^{-1})	36.74	49.57	58.0	7.66	6.98	853.0	73.86
Mn_T (mg kg^{-1})	92.99	305.4	296	33.75	30.19	10554	577.1
Al_T (mg kg^{-1})	22676	25664	9624	433.2	561.1	14966	2307
Fe_T (mg kg^{-1})	9486	21284	135157	1855	169.8	12081	2061
Al_o (mg kg^{-1})	4275	2003	563.0	178.3	315.0	8323	273.0
Fe_o (mg kg^{-1})	2333	1239	41860	171.0	74.02	4233	322.2

X_e: exchangeable concentration of the element; X_T: total concentration of the element; Al_o, Fe_o: extracted with ammonium oxalate

Infrared spectroscopy

In addition to the parameters commented above, some additional characteristics (including the main functional groups present in each material) were determined by infrared spectroscopy on a FTIR-Bomen MB102 equipment (ABB, Zürich, Switzerland). The spectra were obtained by transmittance using KBr pellets, performing determinations in the region between 400 and 4000 cm^{-1}, with a resolution of 4 cm^{-1}.

Forest soil

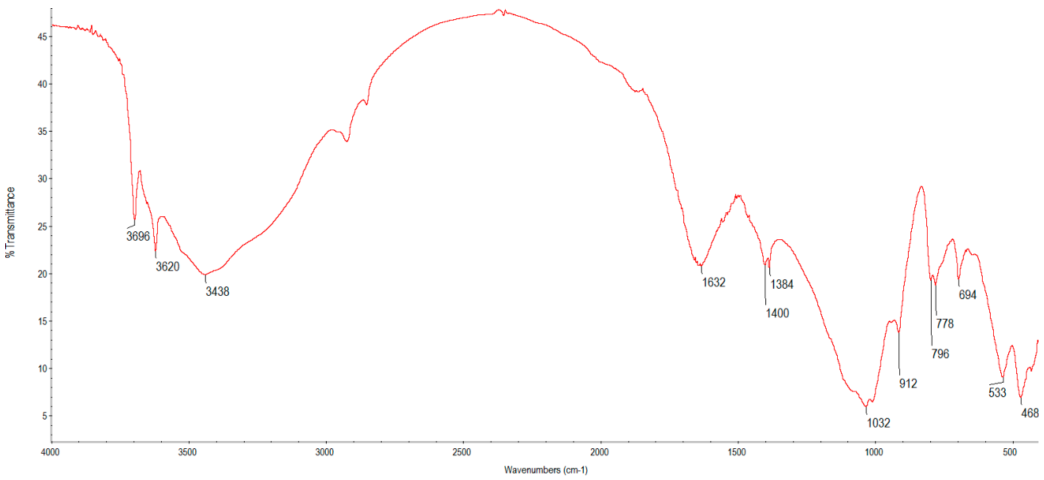

Figure S1. Infrared spectrum of forest soil

The band at 3696 cm^{-1} can be related to the presence of kaolinite, with bands at 3621 and 912 cm^{-1} being typical for clay minerals (Dlapa et al., 2013). Saikia and Parthasarathy (2010) also indicate that a typical spectrum of kaolin should present bands at 3697 and 3620 cm^{-1} (among other). Taking into account that indicated by Haberhauer and Gerzabek (1999), the band at 3438 cm^{-1} would be due to stretching vibration of bonded and non-bonded hydroxyl groups, the one at 1632 would be related to C=O vibrations of carboxylates and aromatic vibrations, although it could be related to H-O-H bending of water (Saikia and Parthasarathy, 2010). Tinti et al. (2015) indicate that a band at approximately 1400 cm^{-1} is present in different clay materials, whereas a band at about 1380 cm^{-1} would be due to coordinatively bound water. As per Margenot et al. (2017), a single broad peak at 1030–1010 cm^{-1} can be related to the 2:1 layer silicates. Haberhauer and Gerzabek (1999) also indicate that a band at about 1050 would be indicative of polysaccharides, or of Si-O vibrations of clay minerals, and bands at about 780, 690 and 540 cm^{-1} would be due to clay, quartz minerals or other inorganic materials.

Vineyard soil

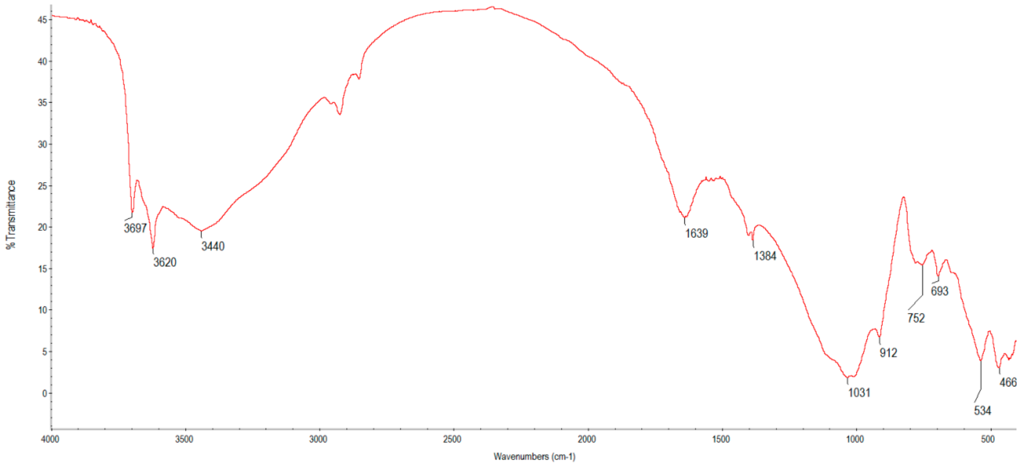

Figure S2. Infrared spectrum of vineyard soil

As in the case of the forest soil, the band at 3697 cm^{-1} would be related to the presence of kaolinite, with this band and those at 3620 and 912 cm^{-1} being typical for clay minerals (Dlapa et al., 2013). The band at 3440 cm^{-1} would be due to H-O-H stretching of adsorbed water (Saikia and Parthasarathy, 2010), whereas the band at 1639 could be related to H-O-H bending of water (although it could be related to C=O vibrations of carboxylates and aromatic vibrations -

Haberhauer and Gerzabek, 1999). As per Sila et al. (2016), a band at about 1639 cm^{-1} would be due to the clay mineral montmorillonite. Tinti et al. (2015) indicate that a band at about 1380 cm^{-1} would be due to coordinatively bound water. Saikia and Parthasarathy (2010) indicate that a band at about 1031 cm^{-1} could be assigned to Si-O stretching of clay minerals. As per Margenot et al. (2017), a single broad peak at 1030–1010 cm^{-1} can be related to the 2:1 layer silicates. The vineyard soil also showed bands at 693, 534, and 466 cm^{-1}, coincident or very close to bands present in the forest soil, and again some of them could be related to that indicated by Haberhauer and Gerzabek (1999): specifically, bands at about 690 and 540 cm^{-1} would be due to clay, quartz minerals or other inorganic materials.

Pyritic material

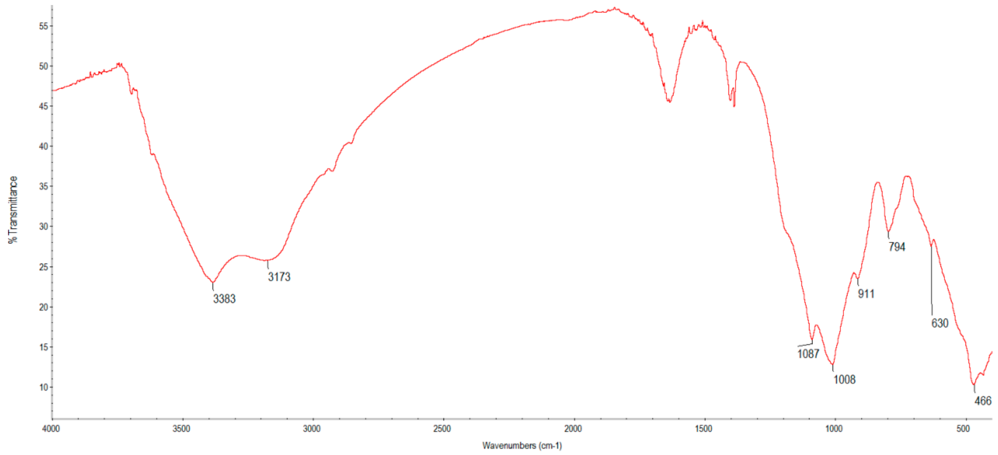

Figure S3. Infrared spectrum of pyritic material

In the pyritic material, the band at 3383 may be related to O–H and N–H stretching, and H-bonded OH (Tinti et al., 2015; Margenot et al., 2017), the band at 1087 cm^{-1} may be due to S-O bonds, or to phosphate (Pavia et al., 2010). Alejano et al. (2014) indicated that characteristic SO functional group bands (corresponding to sulfate), placed at about 474, 512, 871 and 977 cm^{-1}, can be detected both in non-altered and altered pyrite, whereas a band at about 3600 cm^{-1} can be related to OH groups in coordination with metals, which would be due to iron hydroxides (frequent in altered pyrite).

Fine mussel shell

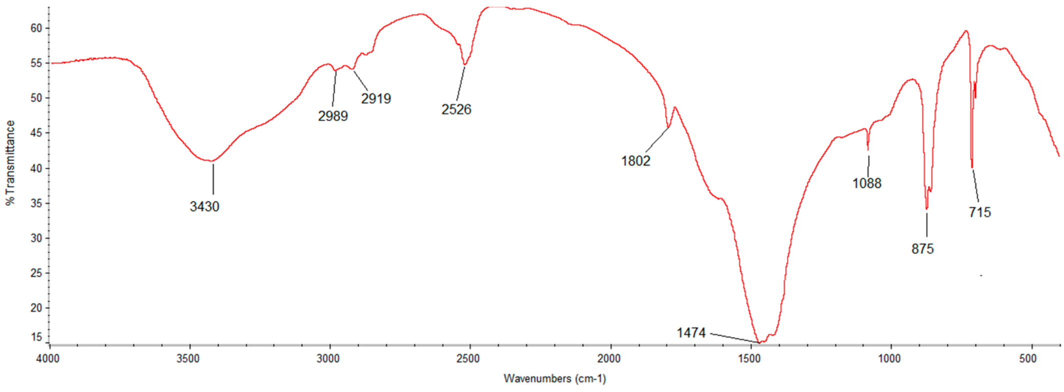

Figure S4. Infrared spectrum of fine mussel shell

The band at 3430 cm^{-1} may be related to N-H bonds, and at 2989 and 2919 cm^{-1} to C-H groups. At 2526 cm^{-1} it may be related to the presence of carbonate groups (Movasaghi et al., 2008; Smidt and Meissl, 2007). The band at 1802 cm^{-1} may be related to the presence of C=O

bindings in acids (Pavia et al., 2010). At 1474 cm^{-1} may be due to CH_2- bonds, and at 1088 cm^{-1} to S-O bonds, or to phosphates (Pavia et al., 2010). The band at 875 cm^{-1} could be due to C-O bonds in carbonates, and at 715 cm^{-1} to N-H bonds (Movasaghi et al., 2008; Smidt and Meissl, 2007).

Pine bark

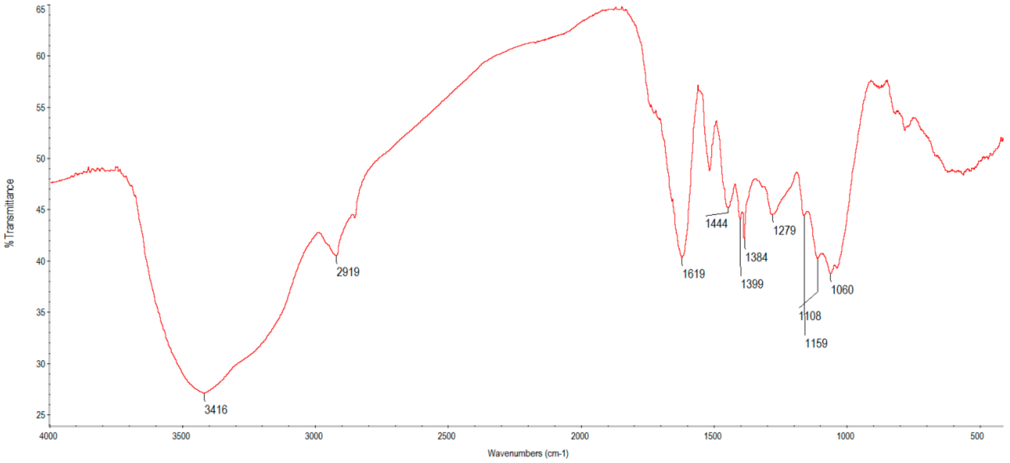

Figure S5. Infrared spectrum of pine bark

In the case of pine bark, FTIR spectrum shows details very similar to those previously found by Brás et al. (2004). Specifically, the band at 3416 cm^{-1} would be due to the bond between the oxygen and the hydrogen stretching vibration; the one at 2919 would correspond to the stretching C-H bond in the aromatic and aliphatic structures; the one situated at 1619 would be due to the aromatic C=C skeletal vibrations; the band at 1444 would be caused by C-H deformation; and the one at 1159 would be in relation to the asymmetric stretching of C-O-C in cellulose and hemicellulose (Brás et al., 2004). Fackler et al. (2010) indicate that the band at 1108 may correspond to ring asymmetric valence vibration of polysaccharides, whereas the band at 1060 may be due to C_3–O_3H valence vibration, mainly from polysaccharides.

Oak ash

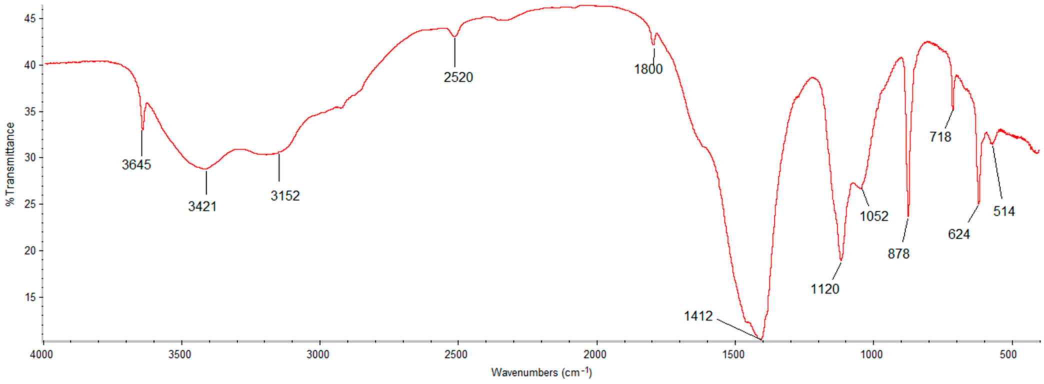

Figure S6. Infrared spectrum of oak ash

The band at 3645 cm^{-1} can be related to O-H stretching for some primary alcohol structures (Pavia et al., 2010). The band in the 3421 cm^{-1} region can be attributed to O-H and N-H bonds, due to the presence of hydroxyl, carboxyl, amine and amide groups (Movasaghi et al., 2008; Smidt and Meissl, 2007; Pavia et al., 2010). The band at 3152 cm^{-1} may be related to the stretching of N-H groups of amines and primary and secondary amides (Pavia et al., 2010). Stretching at 2520 cm^{-1} may be related to O-H bonds in carboxylic acids (Pavia et al., 2010) or to carbonates (Smidt and Meissl, 2007). The band at 1800 cm^{-1} may be related to the presence of

C=O binding in acids (Pavia et al., 2010). At 1412 cm^{-1}, to the presence of nitrates, and C-N, N-H and C-H bonds (Movasaghi et al., 2008; Smidt and Meissl, 2007) or also to folding deformation of $-CH_3$ in alkane groups. The band at 1120 cm^{-1} may be related to the presence of fluoride, C-N-bond amines, or C-O bonds of alcohols, or carboxylic acids (Pavia et al., 2010). It may also be related to C-O-C, C-O, or C-O-P bonds (Movasaghi et al., 2008; Smidt and Meissl, 2007). The band at 1052 cm^{-1} can suggest a C-O bond, phosphate groups (Movasaghi et al., 2008; Smidt and Meissl, 2007) and sulfoxides (Pavia et al., 2010), whereas the band at 878 cm^{-1} can be due to C-O bonds in carbonates. The band at 718 cm^{-1} can be due to N-H bonds, at 624 cm^{-1} may be due to S-O bonds, and at 514 cm^{-1} may be C=C bonds (Movasaghi et al., 2008; Smidt and Meissl, 2007).

Hemp waste

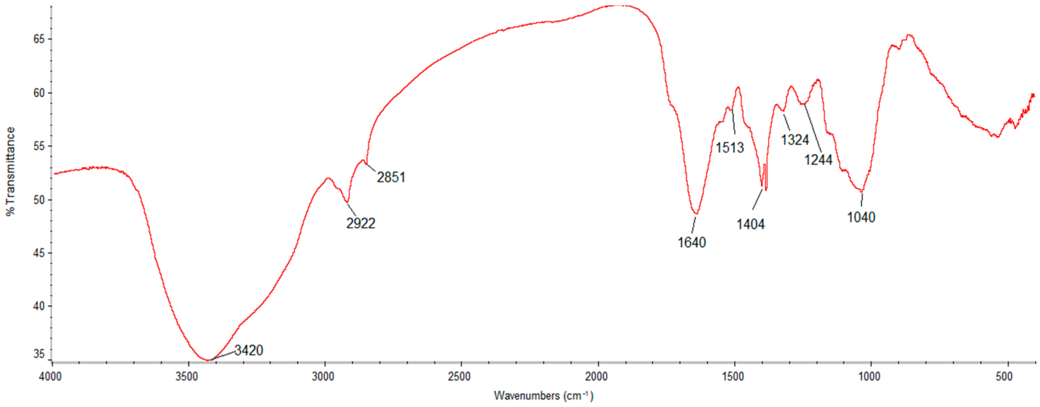

Figure S7. Infrared spectrum of hemp waste

The wide band in the 3420 cm^{-1} region can be attributed to stretching vibration of the O-H bond, suggesting the presence of hydroxyl groups (OH^-) found in cellulose, lignin and water (Smidt and Meissl, 2007; Tarley and Arruda, 2004; Rubio et al., 2013). The bands at 2922 and 2851 cm^{-1} can be related to the stretching vibration of C-H and CH_2 of alkane and aliphatic acids groups (Movasaghi, et al., 2008), also found in cashew nut shell (Coelho et al., 2014). The band at 1640 cm^{-1} can be attributed to C=C bonds in alkenes, or to C=O bonds in amides (Pavia et al., 2010). The band at 1513 can be related to a CH bond in a phenolic ring in lignin (Movasaghi et al., 2008). A folding $-CH_3$ deformation can be found at 1404 cm^{-1}, relating to alkane groups (Pavia et al., 2010). According to these authors, C-N bonds corresponding to aromatic amines can be found in the region 1324 cm^{-1}. The stretching in the frequencies 1244 and 1040 cm^{-1} can be attributed to the presence of asymmetric phosphate groups, C-O of carboxylic acids, and C-O-C, C-O, C-O-P of polysaccharides (Movasaghi et al, 2008; Smidt and Meissl, 2007; Pavia et al., 2010).

References

1. Alejano, L.R., Perucho, A., Olalla, C., Jiménez, R. 2014. Rock Engineering and Rock Mechanics: Structures in and on Rock Masses. CRC Press. London, 372 pp.
2. Álvarez, E., Fernández-Sanjurjo, M.J., Núñez, A., Seco, N., Corti, G. 2012. Aluminium fractionation and speciation in bulk and rhizosphere of a grass soil amended with mussel shells or lime. Geoderma 173/174, 322–329.
3. Brás, I., Teixeira-Lemos, L., Alves, A., Pereira, M.F.R. 2004. Application of pine bark as a sorbent for organic pollutants in effluents. Management of Environmental Quality: An International Journal 15(5), 491–501.
4. Chatterjee, A., Lal, R., Wielopolski, L., Martin, M.Z., Ebinger, M.H. 2009. Evaluation of Different Soil Carbon Determination Methods. Critical Reviews in Plant Science 28, 164–178.

5. Coelho, G.F., ConÇalves Jr., A.C., Tarley C.R.T., Casarin, J., Nacke, N., Francziskowski, M.A. 2014. Removal of metal ions Cd (II), Pb (II) and Cr (III) from water by the cashew nut shell *Anarcadium occidentale* L. Ecol. Eng. 73, 514–525.
6. Dlapa, P., Bodí, M.B., Mataix-Solera, J., Cerdà, A., Doerr, S.H. 2013. FT-IR spectroscopy reveals that ash water repellency is highly dependent on ash chemical composition. Catena 108, 35–43.
7. Fackler, K., Stevanic, J.S., Ters, T., Hinterstoisser, B., Schwanninger, M., Salmén, L. 2010. Localisation and characterisation of incipient brown-rot decay within spruce wood cell walls using FT-IR imaging microscopy. Enzyme and Microbial Technology 47(6), 257–267.
8. Haberhauer, G., Gerzabek, M.H. 1999. Drift and transmission FT-IR spectroscopy of forest soils: an approach to determine decomposition processes of forest litter. Vibrational Spectroscopy 19(2), 413–417.
9. Kamprath, E.J. 1970. Exchangeable aluminium as a criterion for liming leached mineral soils. Soil Science Society of America Proceedings 34, 252–54.
10. Margenot, A.J., Calderón, F.J., Goyne, K.W., Mukome, F.N.D., Parikh, S.J. 2017. IR Spectroscopy, Soil Analysis Applications. Encyclopedia of Spectroscopy and Spectrometry, Third Edition, vol. 2, pp. 448–454.
11. McLean, E.O. 1982. Soil pH and Lime Requirement. In Methods of Soil Analysis, Part 2, Chemical and Microbiological Properties, ASA: Madison, USA, pp. 199–223.
12. Mimura, A.M.S., Vieira, T.V.A., Martinelli, P.B., Gorgulho, H.F. 2010. Utilization of rice husk to remove Cu^{2+}, Al^{3+}, Ni^{2+} and Zn^{2+} from wastewater. Química Nova 33(6), 1279–1284.
13. Movasaghi, Z., Rehman, S., Rehman, I. 2008. Fourier Transform Infrared (FTIR) Spectroscopy of Biological Tissues. Applied Spectroscopy Reviews, 43(2), 134–179.
14. Nóbrega, J.A., Pirola, C., Fialho, L.L., Rota, G., de Campos, C.E., Pollo, F. 2012. Microwave-assisted digestion of organic samples: How simple can it become? Talanta 98, 272–276.
15. Pavia, D.L., Lampman, G.M., Kriz, G.S., Vyvyan, J.R. 2010. Introdução à espectroscopia. 4ª edição, São Paulo, Cengage Learning, 700 pp.
16. Rubio, F., GonÇalves Jr., A.C., Meneghel, A.P., Tarley, C.R.T., Schwantes, D., Coelho, G.F. 2013. Removal of cadmium from water using by-product *Crambe abyssinica* Hochst seeds as biosorbent material. Water Science and Technology 68(1), 227–233.
17. Saikia B.J., Parthasarathy, G. 2010. Fourier Transform Infrared Spectroscopic Characterization of Kaolinite from Assam and Meghalaya, Northeastern India. J. Mod. Phys. 1, 206–210.
18. Sila, A.M., Shepherd, K.D., Pokhariyal, G.P. 2016. Evaluating the utility of mid-infrared spectral subspaces for predicting soil properties. Chemometrics and Intelligent Laboratory Systems. 153, 92–105.
19. Smidt, W., Meissl, K 2007. The applicability of Fourier transform infrared (FT-IR) spectroscopy in waste management. Waste Management 27, 268–276.
20. Sumner, M.E., Miller, W.P. 1996. Cation exchange capacity and exchange coefficients. In Methods of Soil Analysis, Part 3, Chemical Methods, ASA: Madison, USA, pp. 437–474.
21. Tan, K.H. 1996. Soil sampling, preparation, and analysis. Marcel Dekker: New York, USA.
22. Tarley, C.R.T., Arruda, M.A.Z. 2004. Biosorption of heavy metals using rice milling by-products. Characterisation and application for removal of metals from aqueous effluents. Chemosphere 54, 987–995.

23. Tinti, A., Tugnoli, V., Bonora, S., Francioso, O. 2015. Recent applications of vibrational mid-Infrared (IR) spectroscopy for studying soil components: a review. Journal of Central European Agriculture 16(1), 1–22.
